# A Flurry of Folding Problems: An Interview with Susan Lindquist

**DOI:** 10.1371/journal.pgen.1002076

**Published:** 2011-05-12

**Authors:** Jane Gitschier

**Affiliations:** Department of Medicine and Pediatrics, University of California San Francisco, San Francisco, California, United States of America

**Image 1 pgen-1002076-g001:**
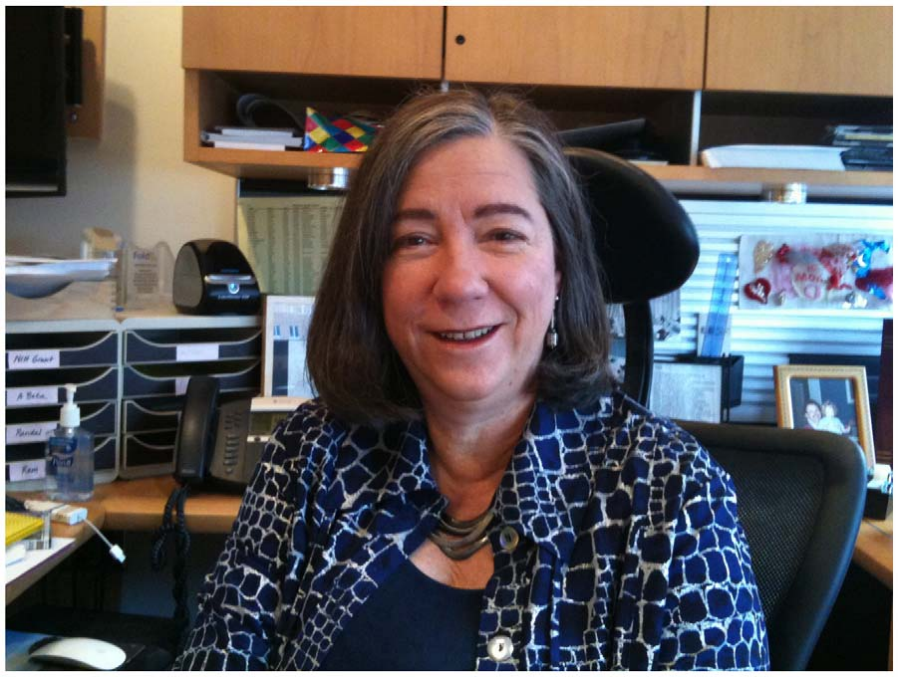
Image 1. Susan Lindquist

Susan Lindquist and I had been trying to intersect for an interview for about 5 years. Lindquist (Image 1), who is currently on the faculty at MIT's Whitehead Institute, is well-known for her work on protein chaperones and stress responses. Her interests grew out of thesis research in the early 1970s in Matt Meselson's lab, where she developed a strategy to study the heat-shock response in cultured cells of *Drosophila*, an approach that complemented observations on transcriptional puffs on polytene chromosomes. In the subsequent 20 years at the University of Chicago, Susan continued to pursue the biochemical and cell-biological properties of heat-shock proteins (HSPs), which assist with a host of protein-folding problems. In 1994, she found herself immersed in a mystery of non-Mendelian inheritance when Yury Chernoff called to say that one such protein, Hsp104, controlled the [PSI+] phenotype. This swept her from stress tolerance to prions and then on to problems of protein folding in cancer and neurodegeneration. I was eager to understand Susan's work and gain her perspective on how chaperones and remodeling proteins promote rapid evolutionary change in response to environmental stresses, a neo-Lamarkian view of biology.

And thus it was that during a cold but sunny January in San Francisco, as I watched the nightly reports of snowstorms in the Northeast, I impulsively decided *now* was the time to visit Susan: I had to be part of the climatic happening. Having grown up in Pennsylvania where snow-day school closures were cherished, and having lived at MIT during the great blizzard of 1978, I diagnosed myself with snowdrift-withdrawal and sought a hit of the fluffy stuff. I managed to fly into and out of Boston easily, intercalating my travel between a series of storms, yet immersing myself in flurries and new-fallen blankets while I was there.

We started our morning interview a bit later than planned, as traffic in Boston was snarled, and Susan began by showing me the picture of her with President Barack Obama in the White House, where she was recently awarded the National Medal of Science.

**Gitschier:** I watched the ceremony online!

**Lindquist:** You did? How sweet of you. It was unbelievable.

**Gitschier:** Let's start with that.

**Lindquist:** Right before this picture was taken I shook his hand and said, “I'm so glad I'm getting this from you and not the other guy.”

**Gitschier:** And what did he say?

**Lindquist:** He smiled. After the ceremony, we had some back and forth banter and it was charming. I said that I brought good wishes from all the scientists I knew, and said that we were really grateful for all that he was doing not just for science, but for the world. And he said, “Yeah, I poll well with scientists.”

**Gitschier:** Did you just want to throw your arms around him?

**Lindquist:** I did! I've never been struck like that before. I really felt like I was in the presence of a great man.

**Gitschier:** Had you known him at the University of Chicago?

**Lindquist:** No. I have a good friend who played basketball with him, though!

**Gitschier:** I know that for the human geneticists in Chicago, it was Obama fever during the campaign and election. I must say the award ceremony was so moving.

**Lindquist:** I thought the remarks he made about how science matters at the highest levels were important. But his decision to bring the high school science fair winners from all over the country to the White House really got me. Can you imagine the thrill of the lifetime it would be for a kid like that? And he loved it. He was chatting with them all, wanted to know what they were doing.

**Gitschier:** It was a big day for prions; I noticed that [Stan] Prusiner also won the Medal.

**Lindquist:** Yes. My citation was mostly for other things, but the role of prions in biology is beginning to explode. Prusiner, Eric Kandel, and I are all going to be speaking at a symposium in honor of Oliver Smithies.

**Gitschier:** Let's talk about the *Aplysia* work with Eric Kandel. You and he had a paper together on CPEB [cytoplasmic polyadenylation element binding protein]. You guys had postulated a few years ago that an amyloid form of this protein aggregated in synapses and could be important for solidifying neuronal connections. Has that gone anywhere?

**Lindquist:** We have a paper in press in *PNAS* where we show that CPEB really does function as a prion. Not proved in neurons yet, but we assembled the fibers *in vitro* with purified recombinant protein, took the cell walls off of yeast, and transformed them from the inactive CPEB state to the active state. So the prion conformation alone switches the protein to a stable active state. It is a cellular mechanism for memory.

**Gitschier:** When you say “active state”, what do you mean?

**Lindquist:** For many of the prions that have been looked at, when they convert to self-templating amyloids, they lose their normal activity. For example, the first prion we worked on is called [PSI+]; it is the amyloid form of SUP35, a translation termination factor. Only a small section of the protein does the aggregation.

**Gitschier:** And the rest of it is dangling out.

**Lindquist:** Right. And when it is converted to the amyloid, SUP35 is sequestered from the ribosome, so now ribosomes don't terminate [translation] when they should. They read through stop codons. But you could imagine the amyloid assembly, with its functional domain dangling out, could also provide a scaffold—a high local concentration of that subunit—that could recruit other factors that need to function with it.

**Gitschier:** Let's talk about the use of the word prion, which was originally coined for a very specific situation in infectious neurodegenerative diseases like scrapie, mad cow disease, and Creutzfeld-Jacob disease. So using the term prion for these other situations—how far are we going to go with this? How was this word originally adapted for yeast?

**Lindquist:** So that was Reid Wickner's terminology, and he based it upon the thought that these heritable phenotypes were due to some sort of a self-perpetuating state that was based on protein.

**Gitschier:** This is his 1994 *Science* paper where he first described [URE3] as a prion and that it was actually an altered protein product of the URE2 gene.

**Lindquist:** Yeah. It could have been a self-perpetuating auto-phosphorylation, protease-activation self-degradation, it could have been all sorts of things.

**Gitschier:** Oh really? You mean he appropriated that term for all of those uses?

**Lindquist:** Yes. In fact, he's published a paper in which a self-perpetuating change in protein cleavage causes a heritable change in phenotype. It's a very clever paper, I think, and not properly appreciated.

But, anyway we found that SUP35 is a self-templating amyloid. The soluble protein can just sit there for hours and doesn't do anything, but if you add just a smidgeon of the amyloid form, it can template the soluble protein into fibers very efficiently. That sequesters it out of solution. So that's what gives it this bi-stable state. That is, it can exist stably as a non-prion or as a prion.

**Gitschier:** So presumably this is a highly cooperative kind of thing.

**Lindquist:** Yes. It turns out to be surprisingly like the PrP protein.

I think that this is where the more we learn about stuff it's not as easy to use simple labels. The same thing has happened with chaperones. The chaperone's initial definition was nice and simple: proteins that bind to other proteins in the immature state, where they are prone to making inappropriate liaisons with other proteins, and it prevents them from doing that. And then when the protein is matured it leaves them alone. Beautiful definition that is really evocative of its function, but now we know that a lot of proteins are stably complexed with chaperones and chaperones can even be part of the functional state. Definitions blur because biology doesn't fit into little boxes. And people get into very big arguments about whether or not you should call this a prion or that a prion.

**Gitschier:** Let's talk about curing prion infections with guanidine hydrochloride. You had a paper that suggested that it wasn't doing it directly, by reconfiguring the prion, as I would have expected, but rather that it was affecting the chaperones.

**Lindquist:** That's how we got into this. We were actually working on it well before Reid Wickner's paper came out.

**Gitschier:** Tell me about that.

**Lindquist:** So, I've been working on heat-shock proteins for a long time. All the major proteins that come up after heat shock have to do with the protein-folding problem, handling it in different ways, mainly as chaperones and protein-remodeling factors. The two that we've worked on the most are Hsp90, which is a protein chaperone, and Hsp104, which is a protein disaggregase.

So, the reason that I got into the prion business was that other folks had shown there was a weird genetic factor that had behaved in a lot of weird ways.

**Gitschier:** You're talking about SUP35 gene product and the [PSI+] phenotype?

**Lindquist:** Yeah.

**Gitschier:** OK, so let's back up and talk about Brian Cox and all of his work on [PSI+].

**Lindquist:** Brian Cox is wonderful. He is an unsung hero.

**Gitschier:** He's got papers back to 1965 in *Heredity*—talking about [PSI+] phenotype, about how there is a heritable but non-Mendelian determinant that suppresses certain auxotrophic mutants in yeast.

**Lindquist:** And I talk about Brian, especially to students, because Brian had a whole series of perplexing observations and he published them. He didn't have to understand the molecular mechanism, and he didn't have to publish them in *Nature*, *Cell*, or *Science*, and it was good quality, rigorous, beautiful work on a difficult problem. We have a tendency, and this happens in my lab all the time, to think that unless we really understand something, we don't publish it.

I think it is a shame, because biology is full of odd observations and it's the odd observations that can wind up pulling together two or three things from different places, and then suddenly you understand it. But in any one line of research, you might not be able to understand it.

So Brian Cox set up all of the basics of this. And there were some Russian groups too, and together they set up all the paradigms that then, when you understand the biochemistry, make it completely easy to understand.

**Gitschier:** Did you know Brian Cox prior to 1994?

**Lindquist:** No, though I had seen him give a talk. There were some concurrent sessions at a genetics meeting, and I remember saying I was going to go to his talk because it sounded really interesting, and I remember a group of friends trying to dissuade me from going to the talk. They said, “Oh God, he's been going on about that forever. Such a weird thing, who wants to hear about that?”

But I was always fascinated by weird things, so I figured I'd go to his talk. I also went to a talk that Sue Liebman gave. All these people had shown that the inheritance of that weird phenotype obeyed very specific rules, they were just different from the normal rules of phenotypes based on DNA mutations.

**Gitschier:** Before 1994.

**Lindquist:** Yeah. And Reid had certainly been thinking about these data when he showed similar things for [URE3], leading to the prion hypothesis.

We got into this because Yury Chernoff, who was working on [PSI+], called me and said, “I've been trying to figure out what's controlling the inheritance of this thing and I just did a genetic screen and the only thing that I've come up with that seems to affect it very strongly is Hsp104. Do you know what it [Hsp104] does?”

The reason he called me was that we had been working on these heat-shock proteins and published a nice paper that showed Hsp104 was responsible for thermo-tolerance in yeast. But we had not yet published its molecular mechanism. In fact, I am at my desk, sitting there quite literally with this paper that had just been rejected from *Nature*. And I said, “Yes I do know what it does, it takes apart protein aggregates, but no one will believe me.” Every one of the reviewers had said that it was ridiculous, that it couldn't possibly be true.

**Gitschier:** So the dogma was that chaperones help you fold initially, but they won't disaggregate.

**Lindquist:** Yes. And I had a hard time even getting people in my lab to consider the possibility, but once we did the experiments it was clear that that was what it was doing. So, anyway, if a cell is defective in Hsp104, it never gets rid of heat-induced aggregates.

**Gitschier:** So are these aggregates prions?

**Lindquist:** They can be! You are anticipating the answer. We found that if you knock out Hsp104, you lose the phenotype caused by Sup35, and if you overexpress it, you also lose the phenotype.

We knew that Hsp104 controlled protein aggregation, so we asked whether there was a difference in the physical state of SUP35 protein in [PSI-] and [PSI+] cells. It was an obvious question. And the answer was “Yes!” SUP35 forms large aggregates in [PSI+] cells and Hsp104 gets rid of them.

**Gitschier:** Is that part of this Chernoff paper, or later?

**Lindquist:** It's later. In Yury's paper the cool thing was that just transient expression of Hsp104 was sufficient to cure it. So here we have two new things: transient overexpression of SUP35 gives a heritable new phenotype, and the transient overexpression of Hsp104, a protein disaggregase, switches cells back heritably to the other phenotype.

Everybody thinks of prions as being bad, scary things. But in yeast cells this prion domain has been conserved for about 800 million years. You can take the prion domain off SUP35 and substitute the corresponding sequence from *Candida albicans*, and not only does it form the prion, it is also controlled by Hsp104.

So why might they have this? Without enough soluble SUP35, the ribosome is going to be reading through lots of stop codons. It could be activating pseudogenes that have a stop codon. It could add additional amino acids and alter the folding of the protein. Or change messenger RNA stability because where the ribosome is on the message can cause the message to be stable or unstable.

**Gitschier:** So you're suggesting it's kind of like an SOS thing.

**Lindquist:** Sort of. You might expect to have a bunch of new phenotypes with [PSI+], which might be different in different strains because the stuff that is downstream of stop codons is not highly conserved. So there will be different consequences when you read beyond the stop codon in different strains. We think it provides a survival advantage through phenotypic variation. Many of the phenotypes will be bad, and so what? Typically one in a million cells switches to the prion. If they die, no harm done. But if [PSI+] happens to provide a good phenotype in a bad environment, a few cells in that colony may survive and their genome will survive. And that could also lead to the evolution of new traits with a few additional mutations. So our idea is that this is a bet-hedging strategy for a few cells in the colony to start placing different bets in terms of what phenotypes they want to have.

And what's cool about this is that it allows the organism to acquire a complex trait in a single step. Because you read through lots of stop codons some will be good, some will be bad, but it is combinatorial.

So the logic of this is that the rate at which cells switch into the prion state really should increase with stress. If things aren't so good, they may want to try that bet—flip into trying a new phenotype more often. And we tested that, and that happens. We have a lot of data on this: lots of different stresses will cause the cells to switch into a prion state, or if you're already in the prion state, they'll switch away from it more frequently. And it's all tied up with protein homeostasis. So if the cell is not well-adapted to its environment, it's more likely to have a protein-folding problem, it's more likely a protein will switch to the prion, and it's more likely to induce Hsp104 and switch out of the prion state. Instead of one in a million cells switching, one in ten thousand do.

**Gitschier:** Of course, this is all based on SUP35 prion formation—allowing ribosomes to read through stop codons. Does *Drosophila* have this prion domain on SUP35, for example?

**Lindquist:** No, it's just in fungi. But in my view this switch is part of early evolution and early life, because many different proteins can form amyloid states. We did a screen for new prions: we've now found 25 new ones in yeast.

**Gitschier:** You screened for it in what way—bioinformatics?

**Lindquist:** Bioinformatics and then testing. We had 200 candidates, looking for these weird domains, we tested 100 of them, and 25 are prions. They create all kinds of interesting phenotypes.

**Gitschier:** Enough for 25 more graduate students!

**Lindquist:** Absolutely. One causes the cells to come together and form biofilms, for example. The proteins are enriched in RNA-binding and DNA-binding and signal transducers. So they allow the cell to try out different things and it will be stable and heritable. So we think we have this whole host of capacitors for evolutionary change.

**Gitschier:** Now when you say the word “capacitors”, what do you mean?

**Lindquist:** We first used the term for Hsp90. I think that will be even bigger than prions.

**Gitschier:** OK, glad I asked.

**Lindquist:** What we meant by that is that Hsp90 provides a way for organisms to accumulate a lot of genetic variation that lies hidden in their genomes and then, under conditions of stress, they release that variation and they can acquire new traits. This is a way in which the environment can cause inheritance of new phenotypes. Both are Lamarckian mechanisms: Lamarck said that environment could cause the inheritance of a new trait. It does! Prions do, so does Hsp90.

So the cells are storing genetic variation, like an electrical capacitor stores charge, and there are mechanisms for releasing it suddenly and in combinatorial fashion.

**Gitschier:** I understand the metaphor for prions, but how does it relate to Hsp90? I take it that Hsp90 is a chaperone?

**Lindquist:** Yes, not a remodeling factor like Hsp104.

We were studying Hsp90 in yeast, way back when it first became possible to knock-out a gene in yeast. We found that it's a very abundant protein, but cells normally need only a little bit of it. But they do need all of it when they are stressed. So it's acting as a protein-folding buffer—excess folding capacity that gets used up with stress. And that is when interesting things happen—with environmental stress.

Hsp90 has a very interesting set of client proteins. It was found complexed with steroid hormone receptors as well as oncogenic kinases—but *inactive* receptors and kinases. So it was postulated that Hsp90 is a protein repressor. And this was coming out just when we are sitting there with genetically modified yeast with different levels of Hsp90. So we said OK, let's use our yeast cells as a “living test tube” and put these other proteins in there. If Hsp90 is a repressor, if you reduce levels, the proteins should become more active. And the opposite happened.

For example, Src kinase normally folds back on itself and self-inhibits. And the mutations that activate it knock out that ability to fold back on itself; Src opens up and it's highly unstable. Hsp90 comes along and says, “Let me save you—you are just the kind of protein I want to bind to!” And while Src's bound to Hsp90 it is inactive, but what Hsp90 is really trying to do is helping it to fold, stabilize it, and get it to the membrane so that it can then be active. So this buffer is enabling the mutant kinase to create an immediate new phenotype. This is sort of the opposite of Hsp90's role as a buffer that hides traits. In this case the excess folding buffer potentiates the effects of mutations immediately.

Hsp90 is involved in folding all kinds of meta-stable proteins—kinases, transciption factors, steroid hormone receptors—that are not meant to be fully folded until they get a signal. Imagine that you start to accumulate some mutations. Hsp90 could hide their effects by letting proteins continue to fold. Then when you reduce the buffer, you might create new phenotypes in a different way, because all hell breaks loose.

So in *Drosophila*, if you have Hsp90 heterozygote mutants, they are fine. But we found that depending on the genetic background, an individual fruit fly might have a funny wing or leg or eye. That is, reducing the Hsp90 folding buffer is altering effects of genetic variation that was hidden in the genome and allowing new phenotypes to be manifested.

Then we found that by just increasing the growth temperature, you also see the same traits; by raising the temperature, you create protein homeostasis stress and deplete the Hsp90 buffer.

I know of no mutation that is as pleiotropic as this—we saw hundreds of different phenotypes. And these were combinatorial phenotypes. The phenotype depended on lots of different genetic variants coming together to make a phenotype.

And then we saw the same thing in *Arabidopsis*—all kinds of phenotypes that depended on Hsp90. Things growing like a vine in agar, hairy roots, plants growing upside-down, leaves coming out in different ways. Lots of wild stuff. And we saw most of the same phenotypes at higher temperature. And so you are losing and gaining phenotypes with environmental stress.

In both Hsp90 and prions, the role of protein homeostasis interfaces with environmental stress to create new phenotypes. And there are ways for both of those to become heritable.

**Gitschier:** I don't want to miss the opportunity to ask you a few more things before we close. How did you get interested in science?

**Lindquist:** I never expected to become a biologist. My parents expected me to become a housewife.

**Gitschier:** What did your parents do?

**Lindquist:** My mother was a housewife and my father was a carpenter/contractor and then he became a tax accountant after a financial setback.

**Gitschier:** What did your parents make out of your wanting to go to graduate school?

**Lindquist:** They thought it was nuts. I'll never forget my trip back home after my first semester in graduate school. I had a paper to finish. The night before New Year's Eve my parents came back around midnight from a party and said, “Are you still working? When are you going to settle down?” I can still remember the exact words, as though I was doing some kind of ridiculous, frivolous thing. When I first had kids they really expected me to quit, too.

**Gitschier:** When was that?

**Lindquist:** I was lucky not to meet the man I wanted to have children with until I had tenure—Edward Buckbee. And he is the most wonderful person in the world. He has been an unbelievable partner to me. I would not be where I am without him.

Having children is a major intensifier in your life—it's not easy! And having the tenure thing settled and having a little more money at your disposal, those things made having children a lot easier. I have a lab full of people with children, and bless them—I love having all these children in my lab. But for me, I'm glad the circumstances happened to be that I waited a while.

You know, once my parents saw I was successful and that I was doing something that was worthwhile and important, they became extraordinarily supportive. They were wonderful and they were such loving people. And my mother spent the last 3 years of her life living with us, and she was so proud, it was so sweet and cute. She'd brag about me to everyone.

**Gitschier:** She didn't get to see you win this big award.

**Lindquist:** [With tears brimming] That was the one really sad part. It would have been a life-long fulfillment if she could have seen that.[Fig pgen-1002076-g001]

